# Activating the Astrocytes of the Dorsal Raphe Nucleus via Its Neural Circuits With the Medial Prefrontal Cortex Improves Depression in Mice

**DOI:** 10.1155/bn/8890705

**Published:** 2025-01-02

**Authors:** Jingyu Zhao, Yuang Wang, Chunxiao Tian, Jialiang Wang, Feng Chen, Xi Dong, Jiayi Luo, Yuxuan Zhu, Aili Liu, Zengguang Ma, Hui Shen

**Affiliations:** ^1^Laboratory of Neurobiology, School of Biomedical Engineering and Technology, Tianjin Medical University, Tianjin, China; ^2^Institute for Translational Neuroscience, The Second Affiliated Hospital of Nantong University, Nantong, China; ^3^Institute for Translational Brain Research, Fudan University, Shanghai, China; ^4^Laboratory of Neurobiology, School of Basic Medical Sciences, Tianjin Medical University, Tianjin, China

**Keywords:** astrocyte, chemogenetics, depression, DRN, mPFC

## Abstract

Astrocytes are the primary cell type in the central nervous system, responsible for maintaining the stability of the brain's internal environment and supporting neuronal functions. Researches have demonstrated the close relationship between astrocytes and the pathophysiology and etiology of major depressive disorder. However, the regulatory mechanisms of astrocytes during depression remain unclear. The aim of this study is to examine the alterations of calcium signaling of astrocytes in the dorsal raphe nucleus (DRN), the calcium signaling alterations of neurons in both the DRN and medial prefrontal cortex (mPFC), and the alteration of depressive-like behaviors by activation of DRN astrocytes using chemogenetics in chronic social defeat stress (CSDS) mice. The results showed that the intensity of calcium signaling in DRN astrocytes was decreased and the frequency of calcium signaling was lower after CSDS. The activation of DRN astrocytes increased the calcium signaling of the neurons including CaMKII*α* neurons in both DRN and mPFC (via neural circuit between DRN and mPFC). The depressive-like behaviors were improved by activating DRN astrocytes in CSDS mice. Our results suggest that the astrocytes in DRN have an important role in depression and the findings offer new insights for the treatment of depression.

## 1. Introduction

Depression is a neurological disorder characterized by a constellation of symptoms, including persistent sadness, low mood, anhedonia, and abnormal appetite and sleep [1, 2]. The causes of depression are complex and multifaceted, involving genetic, environmental, and psychological factors [3, 4]. The 5-hydroxytryptamine (5-HT) transporter protein is the target of fluoxetine, the most widely used antidepressant today [5, 6]. Although fluoxetine and other monoamine antidepressants have shown some success in treating depression, they have notable limitations [7, 8]. These drugs typically take 3–4 weeks to produce significant effects, do not work for everyone, and have side effects, including an increased risk of suicidal tendencies in some patients [9, 10]. A major reason for these limitations is the incomplete understanding of the complexity and interactions within the brain's nervous system during depression.

The dorsal raphe nucleus (DRN) is the origin of most 5-HT projections in the forebrain [11]. It has extensive projections to the cerebral cortex and plays a crucial role in regulating depression [12]. Besides 5-HT neurons, the DRN contains a significant proportion (estimated at 50%–75%) of neurons that primarily release glutamate and gamma-aminobutyric acid (GABA) and are coexpressed with 5-HT [13, 14]. The medial prefrontal cortex (mPFC) innervates many brain regions and is involved in the high-level control of emotional expression, such as anxiety and depression [15, 16]. Neural circuits between the mPFC and the DRN have been implicated in the pathophysiological mechanisms of depression [17, 18]. However, changes in neural activity in the DRN and mPFC during depression are not fully understood.

Recent findings suggest that neuroglia, particularly astrocytes, are closely related to the pathophysiology of major depressive disorder [19]. Astrocytes and their specific receptors are of pharmacological importance as drug targets [20]. Despite the recognized importance of astrocytes, their regulatory mechanisms in depression are still unclear. Most research on the role of astrocytes has focused on mood-related brain regions such as the prefrontal cortex (PFC) and hippocampus [19]. Recent studies indicate dysfunction in the signal transduction of serotonin and astrocytes in depressive-like states [21]. The calcium signaling changes of astrocytes in the central nervous system are similar to the changes of neuronal membrane potential [22]. Astrocytes can respond to most neurotransmitters and neuromodulators and produce calcium ion (Ca^2+^)–mediated signaling, releasing substances such as glutamate, ATP, D-serine, and neurotrophic factor, which affect the structure and function of neurons [23]. Therefore, astrocyte calcium activity can not only reflect the feedback of astrocytes on environmental regulation but also be an important indicator to measure their regulation of the surrounding environment [24]. However, there are no studies on the regulatory role of astrocytes in the DRN during depression or on the chemogenetic activation of DRN astrocytes in regulating depressive-like behaviors and calcium signaling changes in DRN and mPFC neurons.

To address these gaps, this study explores the relationship between calcium signaling of astrocytes in the DRN and behaviors in a chronic social defeat stress (CSDS) model of depression. We separately performed chemogenetic activation of astrocytes in the DRN of mice to investigate their effects on depressive-like behaviors. Additionally, we recorded the effects of chemogenetic activation on calcium signaling in DRN neurons and examined neural projections from the DRN to the mPFC using neural tracing and neurophysiological methods. Finally, we recorded the effects of chemogenetic activation on neuronal calcium signaling in the mPFC. This research is aimed at deepening our understanding of the effects of DRN astrocytes on depressive-like behaviors and associated neuronal calcium signaling after CSDS depression modeling and at exploring potential mechanisms of DRN astrocytes in depression and treatment.

## 2. Materials and Methods

### 2.1. Experimental Animals

Adult male C57BL/6J mice (aged 8–12 weeks, weighing 20–25 g) were obtained from Charles River Laboratories (License No. SCXK (Jing) 2021-0006, Beijing, China). The mice were housed in an environment where the temperature was maintained at 25°C ± 2°C. They were provided with a 12-h light/dark cycle (lights on at 7:00 a.m. and off at 7:00 p.m.) and had free access to food and water. Upon arrival, the animals were acclimated to the new environment for 1 week before the start of the experiments. Mice in the same cage were randomly assigned to different experimental groups. All experimental procedures were approved by the Animal Care and Use Committee of Tianjin Medical University (approval date: 30 March 2020).

### 2.2. Systemic Animal Model of CSDS

Depression-related stresses are inherently social, involving environmental stresses, life events, and interpersonal setbacks [25]. Therefore, the CSDS model in mice is a superior model for studying depression, as it more accurately replicates the depression process caused by social factors, leading to long-lasting behavioral and biological changes [26]. In our study, individual C57BL/6J mice were subjected to the CSDS protocol for 14 consecutive days. Each day, mice experienced 5–10 min of social confrontation with CD-1 mice, referred to as direct contact stress. After the confrontation, the C57BL/6J mice were placed on the opposite side of an isolation baffle in the experimental cage for 24 h, representing indirect psychological stress. This procedure was repeated daily for 14 days, with each subject C57BL/6J mouse exposed to a different CD-1 mouse each day [27].

### 2.3. Behavioral Tests

Open field testing (OFT) is a method used to assess the locomotor abilities and anxiety levels of mice [28]. An open field box of 40 cm in length, width, and height was utilized, with a camera fixed directly above to capture the entire bottom of the box. Mice were placed in the center of the box, and their movements were recorded for 6 min. The first minute allowed mice to acclimate to their surroundings, while the remaining 5 min was used to evaluate their locomotor activity and anxiety levels. Time spent in the central region of the open field was analyzed to gauge anxiety. Before each experiment, the open field box was wiped and dried with 75% alcohol to eliminate olfactory cues from previous mice. After wiping, we will use a hair dryer to dry the test box for 3–5 min to ensure that the test box will not be affected by a smell.

Tail suspension test (TST) was used to assess behavioral despair in mice. Medical tape was applied 1 cm from the tip of the tail, and mice were suspended from a device suspension bar, with the tip of their tails approximately 50 cm above the ground, placing them in a head-down position. The mice were recorded for 6 min with their ventral sides facing the camera in a quiet environment. The first minute of data was discarded, and the immobility time during the final 5 min was recorded.

Sucrose preference test (SPT) is used to evaluate anhedonia, a key symptom of depression characterized by the inability to experience pleasure. Mice were individually housed in cages with two bottles of 1% sucrose solution for adaptive training over 24 h. The SPT was conducted at 9:00 a.m. for 1 h following 24 h of food and water deprivation. Each mouse had access to two bottles, one containing 200 mL of water and the other 200 mL of 1% sucrose solution. The volumes of sucrose solution (V1) and water (V2) consumed were recorded. Sucrose preference was calculated as V1/(V1 + V2) × 100%.

For the social interaction test (SIT), C57BL/6 mice were placed in an open field with a net cage (6 × 8 cm) on one side. In the first stage, C57BL/6 mice were placed in the open field for 5 min without a target in the cage; in the second stage, a CD-1 mouse was placed in the cage, and C57BL/6 mice were returned to the open field for another 5 min. Time spent in the social interaction area (16 × 12 cm) and the avoidance area (8 × 8 cm) was recorded using behavioral analysis software. The social interaction ratio (SIR) was calculated as the time spent in the interaction zone during the second phase divided by the time spent in the interaction zone during the first phase. Mice with SIR < 1 were considered susceptible to social avoidance, while mice with SIR > 1 were considered resistant, showing social preference.

### 2.4. Stereotaxic Optical Fiber Implantation and Virus Injection

The surgical procedure was conducted following previously described methods [29]. Mice were anaesthetised with isoflurane and placed in a stereotaxic frame (RWD Life Science, Shenzhen, China) with a heating pad to maintain body temperature. The mice were maintained under anaesthesia with 1.5% vol isoflurane/vol oxygen (O_2_) throughout the procedure. Ophthalmic ointment was applied to prevent eye drying. Once the scalp fur had been trimmed, a small craniotomy (0.5 × 0.5 mm) was created using a dental drill. The stereotactic injection brain region coordinates are as follows: DRN: anterior posterior (AP): −4.36 mm, medial lateral (ML): 0.00 mm, and dorsal ventral (DV): −3.00 mm; mPFC: AP: +2.23 mm, ML: 0.15 mm, and DV: −1.95 mm. For Ca^2+^ signal recording, 200–300 nL of AAV2/5-GfaABC1D-GCaMP7j solution (approximately 2 × 10^13^ particles/mL) was injected into the tissue at a depth of 2.95 mm using a glass micropipette. The following viruses were used for experimentation: rAAV-GfaABC1D-hM3D (Gq)-P2A-mCherry, rAAV-hSyn-GCaMP6f-WPRE-hGH PA, rAAV-CaMKIIa-GCaMp6f-WPRE-hGH, rAAV-hSyn-CRE-WPRE-hGH, and rAAV-EF1a-DIO-EYFP-WPRE-hGH. Viruses were purchased from Brain Case Biotechnology Co Ltd, OBiO Technology (Shanghai) Corp Ltd. After each injection, the micropipette was held in place for 5 min to allow for diffusion. Upon removal of the micropipette, an optic fiber (1.25-mm outer diameter (OD), 0.37 numerical aperture (NA); Shanghai Fiblaser), threaded through a 3.5-mm ceramic ferrule, was implanted 0.1 mm above the injection site and secured to the skull using dental cement. Recordings were performed at least 3 weeks postsurgery to allow robust virus expression. To confirm the position of GCaMP6f expression, all mice were transcardially perfused with 4% paraformaldehyde (PFA) in phosphate-buffered saline (PBS) following the behavioral tests. After overnight fixation in 4% PFA, brain samples were sectioned coronally and imaged using a fluorescence microscope (BX51, Olympus).

### 2.5. Patch Clamp Recording

Artificial cerebrospinal fluid (ACSF) was frozen at −20°C until it reached a sand-like ice consistency [30]. The ACSF composition was as follows (in millimolar): 120 sodium chloride (NaCl), 1.25 sodium dihydrogen phosphate dihydrate (NaH_2_PO_4_-2H_2_O), 10 glucose hydrate (C_6_H_12_O_6_-H_2_O), 26 sodium bicarbonate (NaHCO_3_), 2 magnesium sulfate heptahydrate (MgSO_4_-7H_2_O), 2.5 potassium chloride (KCl), and 2 calcium chloride dihydrate (CaCl_2_-2H_2_O), with a pH between 7.4 and 7.6 and an osmolarity between 290 and 295 mOsm. Additionally, 200 mL of ACSF was prepared and prewarmed to 32°C. The blade was precooled in the frozen ACSF. Mice were deeply anaesthetised with isoflurane (3%–5%), the brain tissues were quickly removed, and their brain tissues were rapidly transferred into the precooled ACSF for 1 min. Brain slices (300 *μ*m) containing the mPFC were secured with glue in the lower slot of a vibrating slicer (Vibratome, Leica VT1200S, Germany). The slicer was then filled with frozen ACSF, and its settings were adjusted (slicing speed: 0.07 *μ*m/s, amplitude: 2.00, and slice thickness: 300 *μ*m) before the brain tissue was cut coronally. Brain slices were incubated in ACSF at 32°C for 30 min, followed by 30 min at room temperature. The slices were then transferred to the recording chamber and allowed to equilibrate for at least 10 min before recording. Whole-cell patch clamp recordings of mPFC pyramidal cells were performed using a 700B amplifier (Molecular Devices, United States) and glass microelectrodes (3–5 M*Ω*). The brain slice was perfused continuously at a rate of 2 mL/min using a perfusion pump while being aerated with 95% O_2_/5% carbon dioxide (CO_2_). To record spontaneous excitatory postsynaptic currents (sEPSCs), the intracellular solution consisted of the following components (in millimolar): 130 K-gluconate, 10 KCl, 10 4-(2-hydroxyethyl)-1-piperazineethanesulfonic acid (HEPES), 5 magnesium ion (Mg^2^^+^) ATP, 0.25 ethylenebis(oxyethylenenitrilo)tetraacetate (EGTA), pH was adjusted to 7.2 with potassium hydroxide (KOH), and osmolarity was adjusted to 280–290 mOsm. sEPSCs were recorded at holding membrane potentials of −70 mV. Fifty micromolar Picrotoxin was added in the ACSF perfusate. To record paired pulse ratio (PPR), place a concentric circular stimulation electrode in the fifth layer of mPFC and search for cells with a thickness of about 50 *μ*m in the same cell layer for whole-cell recording. Voltage clamp is at −70 mV, electrical stimulation delay 500 ms, stimulation time 0.1 ms, interval 50 ms, restimulation 0.1 ms, recording time 3 s, sweep interval 27 s, and recording 6 sweeps. The stimulation intensity is based on recording to around 100 pA of excitatory postsynaptic current (EPSC). PPR calculation is as follows: EPSC2/EPSC1.

### 2.6. Calcium Analysis

Start from the time when mice start moving, socializing, and struggling in behavioral tests. The relative fluorescence changes are expressed using Δ*F*/*F*, excluding interference from the viral injection dose and basal fluorescence intensity [29]. Δ*F*/*F* is defined as the ratio of the fluorescence value (*F*) to the fluorescence baseline (*F* baseline), where the *F* baseline represents the average fluorescence level within 10 s before the recorded target behavior (such as action exploration, social interaction, and struggle) begins. The MultiPhotometry.mlapp program in MATLAB was used to perform signal preprocessing (including baseline correction and behavioral signaling extraction) and to compute Δ*F*/*F*. The calcium dynamics of the DRN were evaluated using the Δ*F*/*F* metrics of amplitude, duration, and area under the curve (AUC).

### 2.7. Statistical Methods

The data were expressed as means ± standard deviation (SD). Statistical analysis was conducted using GraphPad Prism software, while Adobe Illustrator 2020 facilitated chart creation. Clampfit, MATLAB (2018a), and MiniAnalysis were utilized to evaluate the frequencies and amplitudes of sEPSCs and PPR. Initially, all data were tested with the Kolmogorov–Smirnov test for normality and confirmed to be normally distributed unless otherwise noted. Paired *t*-tests were used to analyze the amplitude and the AUC of DRN astrocyte calcium signaling. One-way analysis of variance (ANOVA) was employed to compare data among different groups. Statistically significant differences were established at ∗*p* < 0.05, ∗∗*p* < 0.01 and ∗∗∗*p* < 0.001, two-sided.

## 3. Results

### 3.1. CSDS Decreased DRN Astrocyte Calcium Signaling

In this study, we investigated the effect of CSDS depression modeling on the Ca^2+^ levels of astrocytes in the DRN of mice using a multichannel fiber-optic photometer system in combination with GCAMP7J-labelled astrocytes. *T*-tests were used to analyze the changes in the area under the relative fluorescence curve (Δ*F*/*F*) of Ca^2+^ in the two groups.

**Figure 1:**
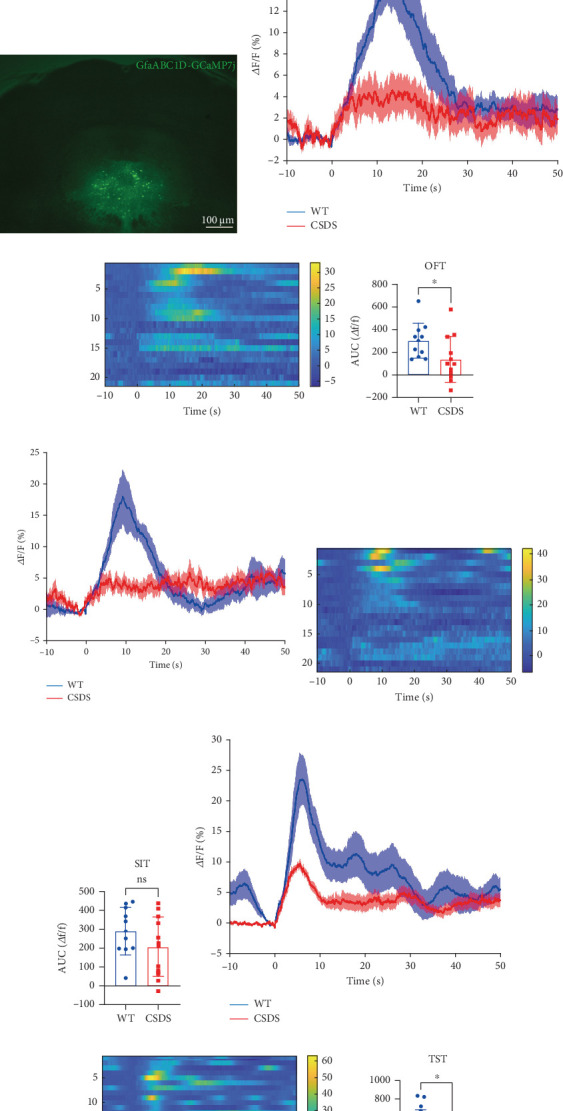
Changes in calcium signaling in astrocytes in the DRN after CSDS modeling. (a) Schematic diagram of the flow of virus injection and calcium signaling recording experiments. (b) Fluorescence image of DRN brain region: expression location of virus AAV2/5-GfaABC1D-GCaMP7j. (c) Calcium signaling Δ*F*/*F* changes of astrocytes of the DRN in OFT. (d) Heatmap of astrocyte calcium signaling Δ*F*/*F* changes of the DRN in OFT. (e) Change in AUC of astrocyte calcium signaling Δ*F*/*F* of the DRN in OFT (blue: control group, red: CSDS modeling group, *T*-test, ⁣^∗^*p* = 0.0417, *n* = 11 trials in six mice). (f) Calcium signaling Δ*F*/*F* changes of astrocytes of the DRN in the SIT. (g) Heatmap of astrocyte calcium signaling Δ*F*/*F* changes of the DRN in the SIT. (h) Change in AUC of astrocyte calcium signaling Δ*F*/*F* of the DRN in the SIT (*p* = 0.1847, *n* = 11 trials in six mice). (i) Calcium signaling Δ*F*/*F* changes of astrocytes of the DRN in the TST. (j) Heatmap of astrocyte calcium signaling Δ*F*/*F* changes of the DRN in the TST. (k) Change in AUC of astrocyte calcium signaling Δ*F*/*F* of the DRN in the TST (⁣^∗^*p* = 0.0161, *n* = 14 trials in six mice).

We first injected the GCaMP virus AAV2/5-GfaABC1D-GCaMP7j [31], which specifically labels astrocytes, into the DRN and then implanted ceramic inserts. After 3 weeks, stable expression was observed in the corresponding brain regions of the mice. Multichannel fiber-optic photometric detection technology was used to record the calcium signaling of astrocytes in the DRN of mice under normal conditions during OFT, SIT, and TST as the wild-type (WT) group. CSDS modeling was performed for 2 weeks after initial recording, and calcium signaling recordings were repeated postmodeling (Figure 1(a)). After the experiment, the injection site of the virus was confirmed using a fluorescence microscope (Figure 1(b)). The experimental results showed that the peak values of Δ*F*/*F* of calcium signaling changes in astrocytes in the DRN during each walking exploration in the OFT under normal conditions were higher than those in mice after CSDS depression modeling. This indicates a decrease calcium signaling in astrocytes in the DRN after depression modeling (Figures 1(c) and 1(d)). Calculation of the area under the Δ*F*/*F* curve for calcium signaling revealed a concomitant decrease in the AUC, suggesting that astrocyte activity was reduced in the depressed state (Figure 1(e)). Following CSDS depression modeling, the Δ*F*/*F* calcium signaling of astrocytes in the DRN showed a decrease in amplitude during the SIT, with the same rate of rise of calcium signaling but a decrease in rise time (Figures 1(f) and 1(g)). However, the AUC of astrocyte calcium signaling did not show statistical difference (Figure 1(h)). We measured the level of despair in mice using TST and observed changes in calcium signaling in astrocytes during the TST. The Δ*F*/*F* calcium signaling of astrocytes in the DRN decreased in amplitude after CSDS depression modeling, and both the rate and rise time of the calcium signaling decreased (Figures 1(i) and 1(j)). This condition resulted in a decrease in the AUC of astrocyte calcium signaling in this brain region (Figure 1(k)).

### 3.2. Chemogenetic Activation of DRN Astrocyte Activity Affected Depressive-Like Behaviors in Mice

**Figure 2:**
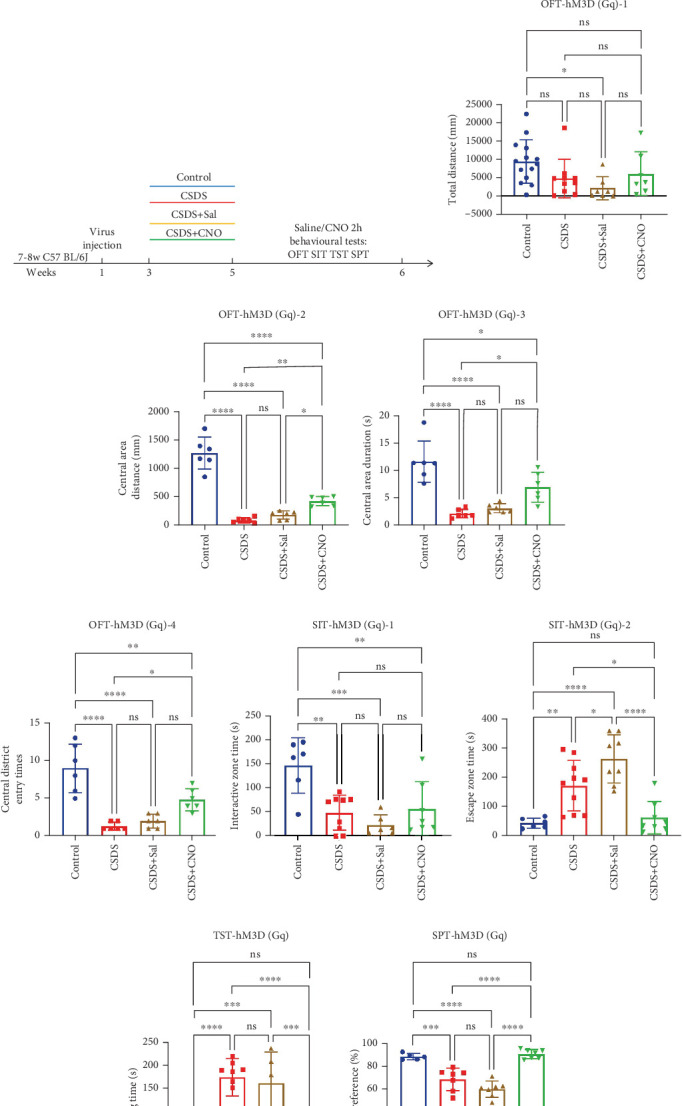
Chemogenetic activation of DRN astrocyte activity induces depression-like behavioral changes in mice. (a) Scheme of chemogenetic virus injection. (b) Fluorescence image of the virus. (c) Experimental process diagram. (d) Chemogenetic activation of DRN astrocytes leading to changes in total distance travelled by mice in OFT (blue: control group, red: CSDS modeling group, brown: CSDS + Sal group, and green: CSDS + CNO group). (e) Chemogenetic activation of DRN astrocytes leading to changes in the distance that mice enter the center area of the open field in OFT. (f) Chemogenetic activation of DRN astrocytes leading to changes in the duration that mice enter the center area of the open field in OFT. (g) Chemogenetic activation of DRN astrocytes leading to changes in the times that mice enter the center area of the open field in OFT. (h, i) Chemogenetic activation resulted in mice being in the interactive zone (h) and escape zone (i) for time changes in the SIT. (j) Chemogenetic activation of DRN astrocytes leads to temporal changes in rocking (resting) mice in the TST. (k) Chemogenetic activation of DRN astrocytes resulted in changes in the proportion of sugar water consumed by mice (the volumes of sucrose solution (V1) and water (V2) consumed were recorded. Sucrose preference was calculated as V1/(V1 + V2) × 100%) in the SPT (⁣^∗^*p* < 0.05, ⁣^∗∗^*p* < 0.01, ⁣^∗∗∗^*p* < 0.001, and ⁣^∗∗∗∗^*p* < 0.0001, *n* = 5–14 mice).

To investigate whether astrocytes in the mouse DRN influence depressive-like behavior in mice, we injected the DRN with either chemogenetically activated virus rAAV-GfaABC1D-hM3D(Gq)-P2A-mCherry (Figure 2(a)). Three weeks postinjection, after stable expression of the virus in the targeted brain regions, we recorded the behavioral characteristics of the mice under normal conditions using the OFT, TST, SIT, and SPT. Following these baseline recordings, the mice underwent a 14-day CSDS depression model. Behavioral changes were recorded after modeling and again 2 h after administration of clozapine-N-oxide (CNO) or saline to the control group (Figure 2(c)). At the end of the experiment, the location of the virus injection was verified by fluorescence microscopy (Figure 2(b)).

The results showed that CSDS modeling reduced the locomotor distance of mice in the OFT. This effect was reversed in the CNO treatment group, where locomotor activity was restored (Figure 2(d)). At the same time, the duration, times, and distance of mice entering the central area in OFT decreased significantly after CSDS modeling, and there was no significant difference in saline injection. After chemogenetic activation of DRN astrocytes, the anxiety levels of mice were improved, and the duration, times and distance of entering the central area were increased (Figures 2(e), 2(f), and 2(g)). In the SIT, chemogenetic activation of astrocytes in the DRN increased the time spent in the social interactive zone (Figure 2(h)) and decreased the time spent in the social escape zone (Figure 2(i)) compared to the control group. In the TST, chemogenetic activation of astrocytes in the DRN decreased immobility time and improved depressive-like behavior in mice (Figure 2(j)). In the SPT, chemogenetic activation of astrocytes of DRN increased the proportion of sucrose–water consumption (Figure 2(k)), indicating a reduction in anhedonia.

### 3.3. Chemogenetic Activation of DRN Astrocyte Activity Affected Mature and CaMKII*α* Neuron Calcium Signaling in DRN

**Figure 3:**
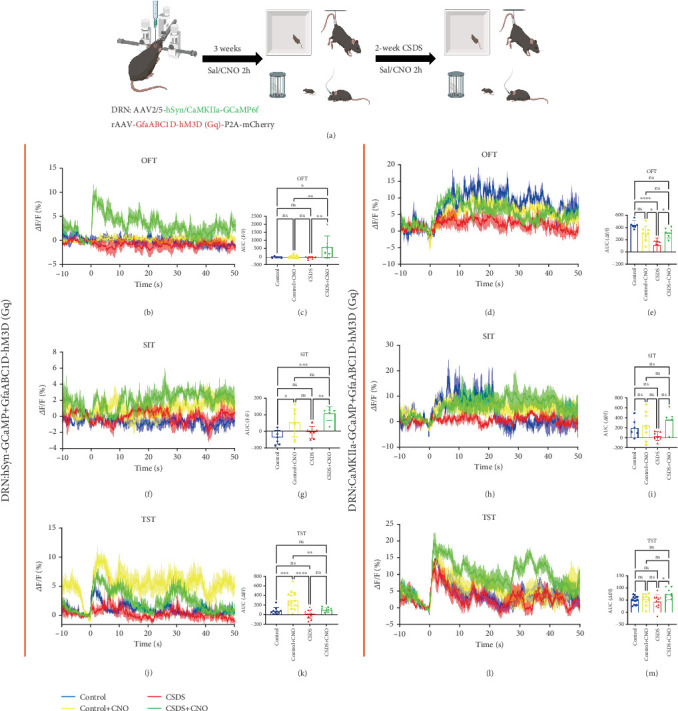
Chemogenetic activation of DRN astrocytes on calcium signaling in mouse DRN mature neurons. (a) Schematic diagram of the flow of virus injection and calcium signaling recording experiments. (b) Changes in calcium signaling in mature neurons of DRN during exploration of mice in an open field box during chemogenetic activation of DRN astrocytes, with Moment 0 as the start moment (blue: control group, yellow: control + CNO administration group, red: CSDS modeling group, green: CSDS + CNO group). (c) Statistical plots of the AUC of mature calcium signaling of the DRN in OFT. (d) Changes in calcium signaling in CaMKII*α* neurons of the DRN during exploration of mice in an open field box during chemogenetic activation of DRN astrocytes. (e) AUC in (d). (f–i) Changes in calcium signaling of mature and CaMKII*α* neurons in the DRN during SIT in mice. (f) Mature; (h) CaMKII*α* and AUC statistics. (j–m) Changes in calcium signaling of mature and CaMKII*α* neurons in the DRN during TST in mice. (j) Mature; (l) CaMKII*α* and AUC statistics (⁣^∗^*p* < 0.05, ⁣^∗∗^*p* < 0.01, ⁣^∗∗∗^*p* < 0.001, and ⁣^∗∗∗∗^*p* < 0.0001, *n* = 10–14 trials in six mice).

To investigate whether astrocytes in the DRN regulate calcium signaling in mature neurons, we injected chemogenetic viruses into the DRN. For activation, we used rAAV-GfaABC1D-hM3D(Gq)-P2A-mCherry. Simultaneously, we injected the GCaMP6f virus rAAV-hSyn-GCaMP6f-WPRE-hGH PA to label mature neurons (regardless of type) or rAAV-CaMKII*α*-GCaMP6f-WPRE-hGH PA, which labels CaMKII*α* neurons, into the DRN of mice. After the injections, a ceramic insert was implanted. Three weeks postinjection, after stable virus expression, we recorded calcium signaling changes in mature neurons under normal conditions and 2 h after CNO administration. These recordings were made during the OFT, TST, and SIT using a multichannel fiber optic photometric detection technique. Following these baseline recordings, mice underwent a 14-day CSDS model. Calcium signaling changes were recorded again postmodeling and 2 h after CNO administration (Figure 3(a)).

In the OFT, chemogenetic activation of DRN astrocytes did not significantly alter calcium signaling in mature neurons of WT mice. However, after CSDS modeling, there was a significant difference in the AUC for calcium signaling, with increased peak and duration observed 2 h after CNO administration (Figures 3(b) and 3(c)). In the SIT, chemogenetic activation of DRN astrocytes increased the peak calcium signaling of mature neurons in the DRN (Figures 3(f) and 3(g)). In the TST, activation of DRN astrocytes led to a decreased peak calcium signaling of mature neurons, with a significant difference in the AUC. After CSDS modeling, calcium signaling of mature neurons in the DRN were elevated, and peak signals were further increased 2 h after CNO administration, showing a significant difference in the AUC (Figures 3(f) and 3(g)). Chemogenetic activation of astrocytes following CSDS depression modeling resulted in a significant increase in CaMKII*α* neuronal calcium signaling in the DRN during the OFT (Figure 3(d)), SIT (Figure 3(h)), and TST (Figure 3(l)). This increase was also reflected in a significant difference in the AUC for this calcium signaling (Figures 3(e), 3(i), and 3(m)).

### 3.4. Neural Projections From the DRN to the mPFC

**Figure 4:**
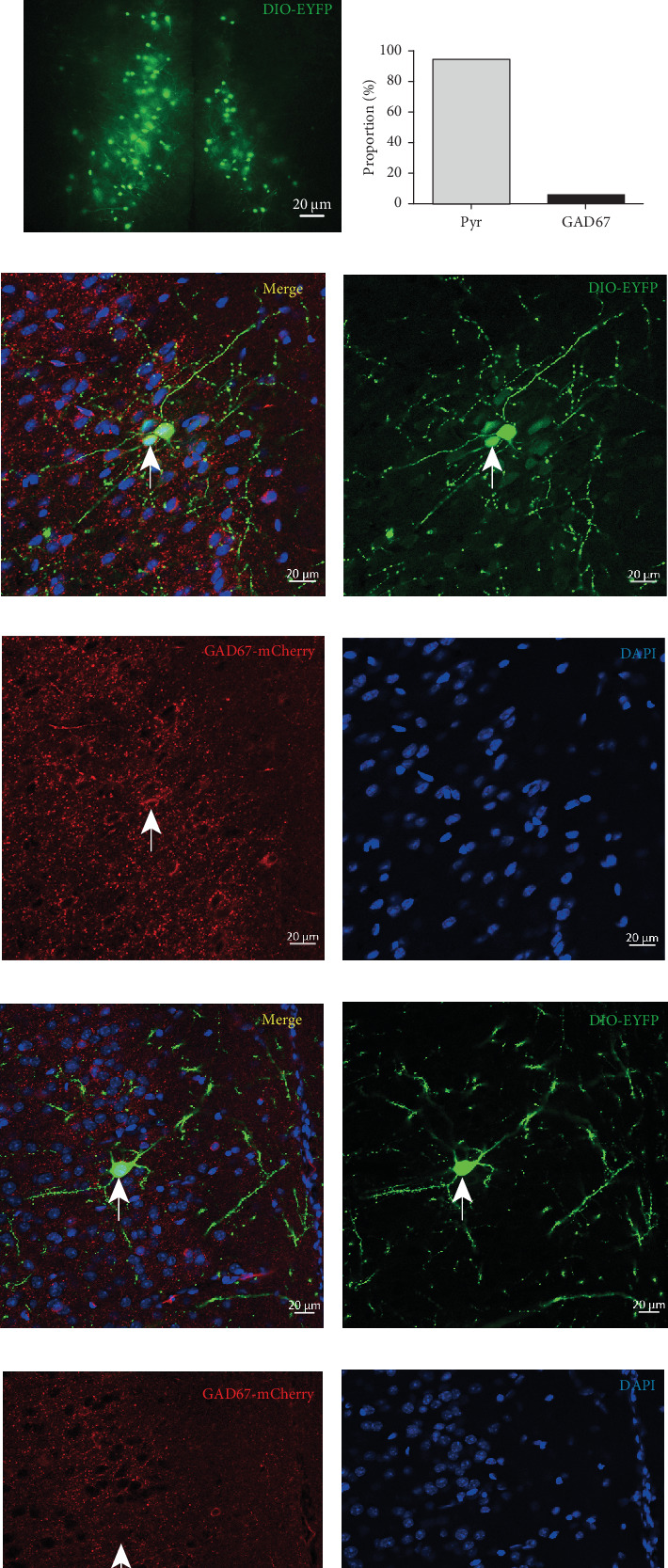
The DRN was projected downstream of the mPFC. (a) Schematic of the DRN and mPFC downstream of neuronal tracer virus injection. (b) Neuronal fluorescence images of mPFC brain regions. (c) Magnified image of neuronal fluorescence image of the mPFC brain region. (d) Statistical graph of the percentage of two types of neurons. (e–h) Schematic of immunofluorescence images of GABAergic neurons (labelled with white arrows, red fluorescence present). (i–l) Schematic of immunofluorescence images of pyramidal neurons (marked with white arrows, no red fluorescence present).

To verify the presence of neuronal projections from the DRN to the mPFC, we used the Cre-loxP system. We injected the DRN of C57BL/6 mice with the Cre virus rAAV-hSyn-CRE-WPRE-hGH, which labels mature neurons, and the mPFC with adenovirus rAAV containing DIO *α*-DIO-EYFP-WPRE-hGH-Ef1 (Figure 4(a)). The fluorescent proteins carried by Cre and DIO are expressed in neurons only when neurons in the mPFC receive projections from the DRN. Consequently, green fluorescence was found in the mPFCs of mice after perfusion and sectioning of the virus-injected brain, confirming that the mPFC receives neuronal projections from the DRN (Figures 4(b) and 4(c)). After verifying these neural projections, we used immunofluorescence staining to classify and count the neurons in the mPFC receiving DRN projections (Figures 4(e), 4(f), 4(g), 4(h), 4(i), 4(j), 4(k), and 4(l)). The results showed that only 5.28% of EYFP-labelled neurons in the mPFC overlapped with GAD67, a marker for GABAergic neurons (Figures 4(d), 4(e), 4(f), 4(g), and 4(h)). This result suggests that DRN primarily sends neuronal projections to pyramidal neurons in the mPFC (Figures 4(i), 4(j), 4(k), and 4(l)).

### 3.5. 5-HT Increased EPSCs of Pyramidal Neurons and Presynaptic Transmitter Release in mPFC

**Figure 5:**
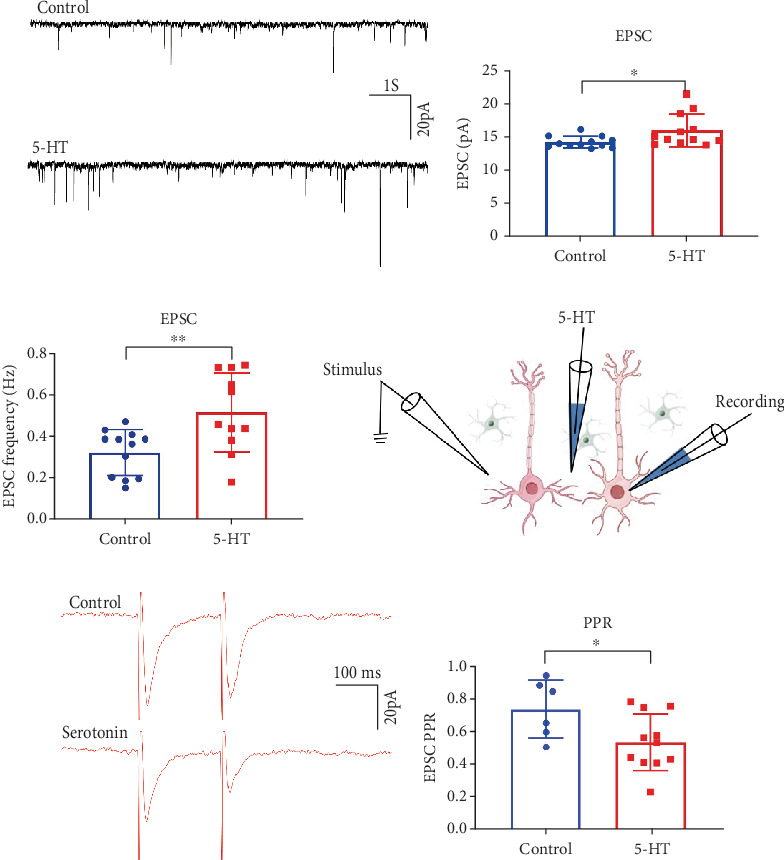
Modulation of mPFC neurons by 5-HT. (a) Changes in sEPSC of pyramidal neurons in the mPFC before and after 5-HT administration (control, 5-HT administration is serotonin). (b) Changes in the amplitude of the sEPSC in pyramidal neurons in the mPFC brain region before and after serotonin administration (control in blue, 5-HT administration group in red, ⁣^∗^*p* = 0.0286). (c) Changes in the frequency of sEPSCs in pyramidal neurons in the mPFC before and after serotonin administration (*n* = 10–12 cells from four mice, ⁣^∗∗^*p* = 0.0066). (d) Schematic diagram of the PPR experiment in the mPFC (recording electrode on the right, stimulation electrode on the left). (e) Changes in PPR of pyramidal neurons in mPFC before and after serotonin administration. (f) Statistical results of changes in PPR of pyramidal neurons in the mPFC before and after serotonin administration (*n* = 6–9 cells from four mice, ⁣^∗^*p* = 0.0372).

Having demonstrated the presence of neuronal projections from the DRN to the mPFC using neural tracing, we sought to verify the modulatory effects of 5-HT from DRN neurons on mPFC neurons. To this end, we employed the patch clamp recording to examine the effects of 5-HT on pyramidal neurons in the mPFC using brain slices. We began by recording sEPSC in mPFC. Upon administering 5-HT (30 *μ*M/mL), we found that both the frequency and amplitude of EPSCs in mPFC pyramidal neurons increased (Figures 5(a), 5(b), and 5(c)). To further investigate whether this increase was caused by enhanced presynaptic transmitter release, we conducted a PPR assay before and after 5-HT administration (Figure 5(d)). The results revealed a decrease in the PPR following 5-HT administration, suggesting that electrophysiological changes caused by 5-HT in the neurons due to an increase in presynaptic transmitter release (Figures 5(e) and 5(f)).

### 3.6. Chemogenetic Activation of DRN Astrocyte Activity Affected CaMKII*α* Neuron Calcium Signaling in mPFC

**Figure 6:**
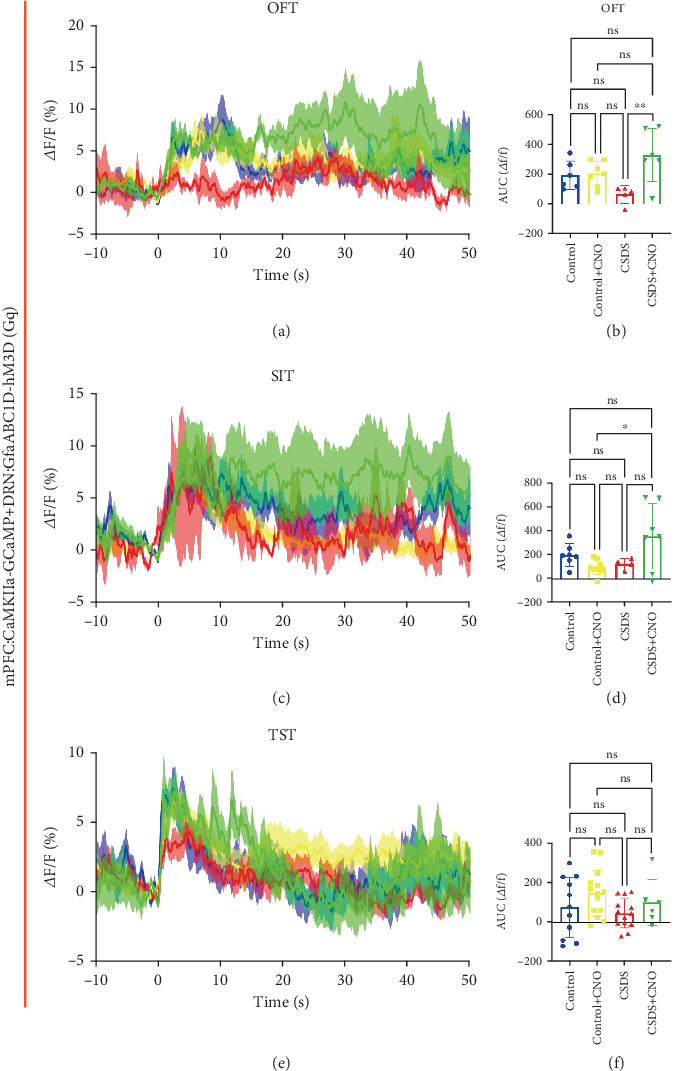
Chemogenetic activation of DRN astrocytes on calcium signaling in mouse mPFC pyramidal neurons. (a) Changes in calcium signaling in pyramidal neurons of the mPFC during exploration of mice in an open field box during chemogenetic activation of DRN astrocytes, with Moment 0 as the start moment (blue: control group, yellow: control + CNO administration group, red: CSDS modeling group, green: CSDS + CNO group). (b) Statistical plots of the AUC of CaMKII*α* calcium signaling in the mPFC in OFT. (c, d) Changes in calcium signaling of pyramidal neurons in the mPFC during SIT in mice and AUC statistics. (e, f) Changes in calcium signaling of pyramidal neurons in the mPFC during TST in mice and AUC statistics (⁣^∗^*p* < 0.05, ⁣^∗∗^*p* < 0.01). (*n* = 6–14 trials in 6 mice).

To investigate whether astrocytes in the DRN regulate calcium signaling in CaMKII*α* neurons in the mPFC, we injected the rAAV-CaMKII*α*-GCaMP6f -WPRE-hGH PA virus, which labels pyramidal neurons, into the mPFC. Results showed that chemogenetic activation of astrocytes following CSDS resulted in a significant increase in the calcium signaling of pyramidal neuronal in the mPFC during the OFT (Figure 6(a)), SIT (Figure 6(c)), and TST (Figure 6(e)). This increase was also reflected in a significant difference in the AUC for this calcium signaling (Figures 6(b), 6(d), and 6(f)).

## 4. Discussion

Depression is a common neurological disorder with a complex pathogenesis, imposing a serious social burden [32]. The onset of action of current antidepressants, such as fluoxetine, a selective serotonin reuptake inhibitor, relies on desensitisation of the DRN 5-HT_1A_R [33]. Additionally, the rapid antidepressant effects of ketamine are linked to both the mPFC and DRN [34]. However, rapid antidepressant studies have primarily focused on 5-HT neurons and receptors in the DRN [35], leaving the changes and roles of DRN astrocytes in depression unclear.

Our findings indicate that after CSDS, mice exhibited decreased calcium signaling in the DRN astrocytes during the OFT, SIT, and TST (Figure 1) [22], similar to the findings in the PFC [36, 37]. Chemogenetic activation of DRN astrocytes, however, improved depressive-like behaviors in these mice (Figure 2). González-Arias et al. found that neuron–astrocyte signaling dysfunction occurs in depressive-like states, emphasizing the important role of astrocyte Ca^2+^ signaling in brain circuits and behavioral homeostasis control [21]. In the state of depression, changes in astrocyte function are mainly reflected in three aspects: neuroimmune status, neuronal signal transduction, and synaptic plasticity [38, 39]. Therefore, chemogenetic activation of DRN astrocytes may also improve depressive-like behaviors in mice by improving neuroinflammatory response [40], providing neurotrophic factors [41], and improving synaptic plasticity [42–44]. This result suggests that DRN astrocytes are less active after depression and play an important role in depression-like behaviors.

In vivo optical fiber photometry can record the changes of calcium signaling of neurons or astrocytes in the mouse brain and analyze the behavior of mice at the same time, which provides great help for neuroscience research [45]. In order to investigate the mechanism of DRN astrocytes in depression, we combined fiber-optic photometry and chemogenetic techniques and found that chemogenetic activated DRN astrocytes and increased calcium signaling in mature neurons in DRN (Figures 3(b), 3(c), 3(f), 3(g), 3(j), and 3(k)). This result indicates that chemogenetic activation of DRN astrocytes improves depressive-like behaviors by regulating neuronal calcium signaling [24]. Due to the presence of multiple types of neurons in DRN and the crucial role of excitatory neuron activity in depression, we further explored the calcium signaling of CaMKII*α* neurons [13, 46]. The results showed that chemogenetic activation of DRN astrocytes increased the calcium signaling of CaMKII*α* neurons in DRN (Figures 3(d), 3(e), 3(h), 3(i), 3(l), and 3(m)). Studies have shown that the excitability of DRN excitatory neurons decreases in depressive states, and activation improves depressive-like behaviors [17, 34]. Therefore, regulating astrocytes may improve depressive-like behaviors by improving the excitability of CaMKII*α* neurons in the DRN brain region.

Due to the extensive projection of DRN to the forebrain and the important role of DRN to the mPFC neural pathway in depression [47], we conducted a study on the neural projection from DRN to mPFC and found that DRN neurons in the brain area can project forward to the mPFC and mainly regulate the pyramidal neurons in mPFC (Figure 4) [34]. DRN is the origin of most 5-HT projections in the forebrain [35]. Besides 5-HT neurons, the DRN contains a significant proportion (estimated at 50%–75%) of neurons that primarily release GABA and glutamate and are coexpressed with 5-HT [13, 14]. In order to further investigate the regulatory effect of DRN on PFC, we conducted a whole-cell patch-clamp experiment, and the results showed that administration of 30 *μ*M/mL 5-HT enhanced the frequency and amplitude of sEPSC discharges in mPFC brain slices and decreased PPR (Figure 5), consistent with previous studies [48]. This result indicates that 5-HT enhances neuronal excitability by increasing the release of presynaptic neurotransmitters.

Finally, based on the above results, we speculate that chemogenetic activation of DRN astrocytes may affect depressive-like behaviors by activating DRN neurons, thereby increasing calcium signaling in mPFC pyramidal neurons [15, 17]. The results showed that chemogenetic activation of DRN astrocytes increased calcium signaling in pyramidal neurons in mPFC (Figure 6). The results indicate that chemogenetic activation of DRN astrocytes will affect the neural pathway from DRN to mPFC, achieving the improvement of depressive-like behaviors.

There are some limitations to our study that should be noted. Firstly, in addition to mPFC, DRN has neural projections to regions such as the amygdala and hippocampus in the brain, and these brain regions all have an impact on depressive-like behaviors [12, 17, 49]. Therefore, more extensive research is needed to explore the neural circuitry of DRN astrocytes in improving depression. Secondly, the combination of chemogenetic activation of DRN astrocytes and SSRIs may reduce the onset time of SSRIs [50, 51]. This may contribute to the study of the antidepressant mechanisms of SSRIs and the development of possible therapeutic strategies for rapid antidepressants.

## 5. Conclusion

In conclusion, our study demonstrated that chemogenetic activation of DRN astrocytes ameliorated depressive-like behaviors in mice. The mechanism may involve a neural pathway from the DRN to the mPFC, providing new insights into the pathogenesis and treatment of depression. We observed that CSDS reduced calcium signaling in DRN astrocytes, suggesting potential pharmacological targets for treating depression. Furthermore, the complex calcium dynamics in the DRN imply that depression is accompanied by intricate physiological and pathological changes. This complexity may explain the prolonged duration and refractory nature of depression to commonly used antidepressants and could aid in exploring more effective treatment methods, such as long-term combinations of different targeted drugs.

## Data Availability

The relevant data in this study are available from the corresponding authors upon reasonable request.
